# Comprehensive analysis of haemostatic profile depending on clinicopathological determinants in breast cancer patients

**DOI:** 10.1042/BSR20171657

**Published:** 2018-03-29

**Authors:** Piotr Rhone, Barbara Ruszkowska-Ciastek, Kornel Bielawski, Alen Brkic, Elżbieta Zarychta, Barbara Góralczyk, Krzysztof Roszkowski, Danuta Rość

**Affiliations:** 1 Clinical Ward of Breast Cancer and Reconstructive Surgery, Oncology Centre Prof. F. Łukaszczyk Memorial Hospital, Bydgoszcz, Poland; 2 Department of Pathophysiology, Faculty of Pharmacy, Nicolaus Copernicus University Toruń, Collegium Medicum in Bydgoszcz, Poland; 3 Department of Oncology, Radiotherapy and Ginecologic Oncology, Faculty of Health Sciences, Nicolaus Copernicus University, Collegium Medicum in Bydgoszcz, Poland

**Keywords:** breast cancer, cancer invasion, clinicopathological determinants, haemostatic profile, hypercoagulable state

## Abstract

Thrombosis is one of the leading causes of mortality in cancer patients. The aim of the study was to evaluate the concentrations and activities of selected haemostatic parameters in the plasma of patients diagnosed with breast cancer (BrCa) and to make an attempt at finding associations with their levels and selected clinicopathological factors; clinical classification, histological grading, and molecular subtype of BrCa. The study involved 145 Caucasian ethnicity women. Eighty-five women aged 45–66 with primary BrCa without distant metastases (M0). Inclusion criteria were as follows: histopathological examination confirming the diagnosis of primary BrCa, without previous radiotherapy and chemotherapy. The control group consisted of 60, post-menopausal women, aged 45–68. Haemostatic profile expressed by concentrations and activities of tissue factor (TF) and its inhibitor (TFPI) as well as concentrations of tissue plasminogen activator (t-PA) and plasminogen activator inhibitor type 1 (PAI-1) were measured applying immunoassay techniques. A significantly higher concentration of PAI-1 was noted in patients with BrCa localized in the left breast. We observed significantly lower activity of TFPI and significantly higher concentration of PAI-1 in the group of patients with invasive ductal carcinoma as compared with invasive lobular carcinoma. A significantly higher concentration of t-PA in patients with pT2 BrCa in relation to pT1 cases was noted. Based on comprehensive analysis of haemostatic profile depending on clinicopathological features, we suggest that haemostatic parameters play crucial roles in invasion and metastases of malignant tumours.

## Introduction

Cancers represent an acquired hypercoagulable state and, unfortunately, the genesis of this phenomenon is both composite and multifactorial and still not fully recognized. General risk of venous thromboembolism (VTE) including deep vein thrombosis (DVT) and pulmonary embolism (PE) is four- to seven-fold enhanced in cancer patients relative to healthy individuals [1,2]. Thromboprophylaxis is not commonly recommended for cancer patients since the risk of VTE is not high enough and cancer patients are more predisposed to bleeding [2].

Cancer cells can disrupt the haemostatic balance by over-releasing of tissue factor (TF), plasminogen activator inhibitor type 1 (PAI-1) as well as increased platelet adhesiveness, which lead to pro-coagulant phenotype [2–5]. Many studies focus on searching the exact pathomechanism(s) of coagulation cascade overactivation. However, the risk of hypercoagulability is variable and is associated with cancer type. Hence, a clinical approach should be tailored according to the localization of the cancer, in as much as pancreatic, ovarian and brain cancers are at a higher risk of danger from thrombosis, intermediate risk is observed in colon and lung cancers, whereas breast and prostate cancers are at lower occurrence of thrombotic diathesis [2,4,5].

Thrombocytes, coagulation and fibrinolysis processes, all of which are parts of the haemostatic system, are known to modulate various mechanisms recognized to control breast cancer (BrCa) development [6]. Full-length TF (flTF), a membrane-associated glycoprotein that is present on the subendothelial layer, is established as the main bridge between the haemostatic process and cancer progression. The fundamental role of subendothelial flTF is initiation of extrinsic coagulation pathway, which plays a crucial role in thrombin generation. Interestingly, tumorigenesis, invasion and metastasis can indirectly be promoted via flTF by means of thrombocyte activation, fibrin deposition and thrombin generation [4,6–8]. flTF tightly regulates cell motility and proliferation as well as stimulating angiogenic switch, and presents anti-apoptotic properties by stimulation of anti-apoptotic proteins production [4,9,10]. However, during recent years another factor has come to light, namely flTF pathway inhibitor-1 (flTFPI-1), the most powerful regulator of TF activity. Primarily recognized for its function within the coagulation cascade. While, TFPI has now been documented as having not only antitumour effects but also provoking apoptosis and suppressing proliferation and cancer invasion [7]. Various studies have shown elevation of both TFPI and TF within cancer patients of different origin and subtypes, as well as in those with BrCa. TFPI are associated with clinicopathological parameters and survival in BrCa, in contrast with the coagulation initiator TF. Unfortunately, the pathomechanism of TFPI functions is as yet unknown [7,11,12].

BrCa originates in mammary epithelial cells and is an extremely multifarious disease with respect to genetic, clinical and molecular aspects. BrCa is a steroid hormone-dependent tumour [8]. The approach to BrCa treatment relies on the availability of clinical and pathological prognostic and predictive determinants. Immunohistochemical (IHC) evolution of the proliferation marker (Ki67), oestrogen receptor (ER), progesterone receptor (PR), and human epidermal growth factor receptor 2 (HER2), is clinically essential as these are markers for both prognosis and type of treatment [8,9]. Upon confirmation of the positive status of ER and PRs, the patient is eligible for hormone treatment, however, when positive HER2 expression is noted this predicts the response to anti-HER2 therapy [12].

Nevertheless, to assess patients with a high risk of recurrence, traditional prognostic factors do not suffice; searching for new markers is still important. Although a major body of research has formed regarding the association between haemostatic parameters and tumour biology, in the clinical setting, poor prognosis and reduced survival rates are often connected with overproduction of TF by tumour cells in BrCa patients [6,7]. At present interactions between TF, TFPI, as well as fibrinolytic profile with clinicopathological features, remain controversial. Better characterization of the relationship between the clinical course of BrCa and haemostatic parameters may provide insight into the underlying risk of hypercoagulability in BrCa patients. In the present case–control study, we aimed to investigate the clinical suitability of selected haemostatic parameters, including TF/TFPI ratio, in BrCa. The interrelationships between concentration and activity of TF and TFPI as well as tissue plasminogen activator (t-PA) and PAI-1 with TNM and histological grading according to Elston–Ellis, molecular subtype of BrCa, ER, PR and HER2, the expression of Ki67 were also tested.

## Methods

### Patients

This was a single-centre, prospective study comprising 145 Caucasian women. The investigation group consisted of 85 females aged between 45 and 66 years (mean age: 55 years) with primary BrCa without distant metastases (M0). Subjects were admitted to the Clinical Ward of Breast Cancer and Reconstructive Surgery, Oncology Centre in Bydgoszcz, Poland. Data collection from the patients ran from November 2015 to June 2017. The median value of patients’ body mass index (BMI) was 25.71 kg/m^2^. The median value of tumour diameter was 1.49 cm. The subjects were prepared for surgical procedure of either breast-conserving surgery (BCS) or modified radical mastectomy (MRM). The cases underwent a comprehensive clinicopathological and post-surgical examination of tumour size (T-status), lymph node (N) status and tumour grade (GI, well differentiated; GII, moderately differentiated; GIII, poorly differentiated). Additionally, molecular determinants including ER status, PR status, E-cadherin expression and HER2 status were obtained from pathology reviews and included in the study (Table 1). Inclusion criteria were as follows: histopathological examination confirming the diagnosis of primary BrCa, without previous radiotherapy and chemotherapy. The exclusion criteria included such conditions as metastatic tumours, other cancers, serious liver, lung, kidney, brain and other organ dysfunctions, autoimmune diseases, recent bleeding or thrombotic events and major surgical history. Additionally, cerebral–vascular diseases, congestive heart failure, overt diabetes mellitus, dyslipidaemia as well as acute and chronic infections were excluded.

**Table 1:** Baseline demographic and clinical characteristics of the study population

Features	Number of patients (%)
**Number of patients:** *n*=85 females	100%
**Median (range)**: 55 years (45–66)	
**Age at diagnosis (years)**	
<55	37 (44%)
≥55	48 (56%)
**BMI (kg/m^2^) according to WHO criteria**	
Normal (<24.99)	40 (47%)
Overweight (≥25–29.99)	30 (35%)
Obese (30 or more)	15 (18%)
**Menopausal status**	
Pre-menopausal	27 (32%)
Post-menopausal	58 (68%)
**MHT administration**	
Oral route of administration	12 (14%)
Transdermal route of administration	4 (5%)
**Parity**	
Nulliparous/1–2 children/>2 children	7 (8%)/60 (71%)/18 (21%)
**History of smoking habit**	
Current smoker/ex-smoker/never smoked	21 (25%)/10 (12%)/ 54(63%)
**Tumour size (T- status)**	
T1 (<2 cm)/ T2 (≥2 cm, but <5 cm)	60 (71%)/25 (29%)
**Nodal status (N)**	
N0/ N1	66 (78%)/19 (22%)
**Histological grading**	
Grade I/II/III	7 (8%)/65 (77%)/13 (15%)
**Histological type of BrCa**	
Invasive ductal carcinoma (IDC)	75 (88%)
Invasive lobular carcinoma (ILC)	10 (12%)
**Molecular type of BrCa**	
Luminal A (*ER+/HER2−/Ki67* < *14%)*	58 (69%)
Luminal B HER *positive* (*ER+/HER2+/Ki67* ≥ *14%)*	8 (9%)
Luminal B HER *negative* (*ER+/HER2−/Ki67* ≥ *14%)*	8 (9%)
Triple-negative *(ER/PR/HER2 negative)*	8 (9%)
HER^+^/non-luminal	3 (4%)
**ER status**	
Positive/negative	74 (87%)/11 (13%)
**PR status**	
Positive/negative	69 (81%)/16 (19%)
**HER2**	
Amplified/normal	11 (13%)/74 (87%)
**E-cadherin**	
Positive/negative	81 (95%)/4 (5%)
**Ki67** cut-off point 14%	
Lower	58 (68%)
Higher	27 (32%)
**Breast surgery**	
BCS	68 (80%)
MRM	17 (20%)
**Localization**	
Left breast/right breast	43 (51%)/42 (49%)

Abbreviations: MHT, menopausal hormonal therapy; Parity, number of full-term pregnancies.

Another 60 women free of breast tumour were selected as the control group and enrolled in the study while waiting for their routine medical visits. The mean age was 52.4 with an age range of 45–68 years. The average BMI value was 25.21 kg/m^2^. The exclusion criteria for the control group was as follows: surgery <1 month, hyperlipidaemia and insulin resistance, overt diabetes, obesity, acute and chronic infections or overt thrombosis. All 145 respondents did not take any medication that could essentially affect the value of the results being anticoagulants, antiplatelet agents, thrombolytic drugs, and nonsteroidal anti-inflammatory drugs.

Hypertension was established according to a systolic blood pressure (BP) value of 140 mmHg or higher and a diastolic BP of 90 mmHg or higher. The BMI was obtained by dividing the weight expressed in kilograms (kg) by the height in square metres (m^2^) and characterized according to the WHO criteria. Obesity is defined as BMI > 30 kg/m^2^. The concentration of fasting glucose, lipid profile and C-reactive protein were measured using specific tests for Automated Immuno-Biochemical Analyzer Cobas®6000, Roche Diagnostics, U.S.A. [13].

All the patients gave their written informed consents. The study was approved by the Bioethics Committee Collegium Medicum in Bydgoszcz; the Nicolaus Copernicus University in Toruń, Poland (reference number: KB/547/2015).

### Estimation of molecular determinants such as ER, PR, Ki67, HER2/neu by immunohistochemistry

The procedure for determination of ER, PR positive status and Ki67 expression of the tumours was based on immunohistochemistry (IMH) and tumour cell nuclei were scored according to pathology guidelines. Additionally, monoclonal mouse antibody for the demonstration of the Ki67 antigen in the specimens (Auto-stainer Link 48, Agilent Technologies, U.S.A.) was used. For the Ki67 proliferation index, we used a 14% threshold as the limit to define high/low proliferative cases. Antibodies VENTANA anti-HER2/neu (4B5) was applied for laboratory semiquantificative detection of the antigen HER2/neu with the use of a VENTANA aperture for staining the IHC microscopic slide (Benchmark Ultra, Roche-Ventana). HER2 was considered positive with a level of (+++) and negative with a level of (–), (+) [13]. However, scores of (++) were taken as equivocal cases, which were further recommended for fluorescence *in situ* hybridization (FISH) confirmation, which detects gene amplification. FISH was performed using a dual HER2/Cep17 probe.

### Sample collection

Venous blood (4.5 ml) for testing concentrations of TF, TF pathway inhibitor (TFPI) α (TFPIα), t-PA, PAI-1 and TF, TFPI activities was collected into cooled tubes (Becton Dickinson Vacutainer® System, Plymouth, U.K.) containing 0.13 mol/l trisodium citrate (final blood anticoagulant ratio 9:1). The standard blood collection protocols were respected; patients had been in a fasting state, after 30 min of rest and after a 12-h overnight fast. The blood samples were immediately mixed and centrifuged at 3000×***g*** at +4°C for 15 min and then frozen at −80°C (as specified by the manufacturer) until assaying within 6 months.

### ELISA

Haemostatic parameters were performed using ELISA with a commercial kit. In all assays, the reaction mixture was added in a 96-well plate. The concentrations of TF, TFPI and PAI-1 were defined using the IMUBIND®Tissue Factor, IMUBIND®TFPI and IMUBIND®Plasma PAI-1 ELISA test Kit 96-Well Plate Assay, Sekisui Diagnostics, LLC, Stamford, CT, U.S.A. respectively. However, the concentration of t-PA was assayed with the AssayMax™ Human t-PA ELISA Kit, Assaypro LLC, St. Charles, MO, U.S.A. Additionally, the plasma activities of TF and TFPI were measured using chromogenic assays, the ACTICHROME®TF and ACTICHROME®TFPI-tests (Sekisui Diagnostics, LLC, Stamford, CT, U.S.A.) respectively.

### Statistical analysis

The Shapiro–Wilk test was used to check the normality of data distribution. Data are presented as medians and interquartile ranges (IQRs). Statistical differences between groups were determined with the non-parametric U-Mann–Whitney rank-sum test. The correlations were sought by the Spearman’s rank method. All probability (*P*) values ≤0.05 were deemed significant. All analyses were performed using the statistical software: Statistica v. 12.0 (StatStoft®, Cracow, Poland).

## Results

**Figure 1:**
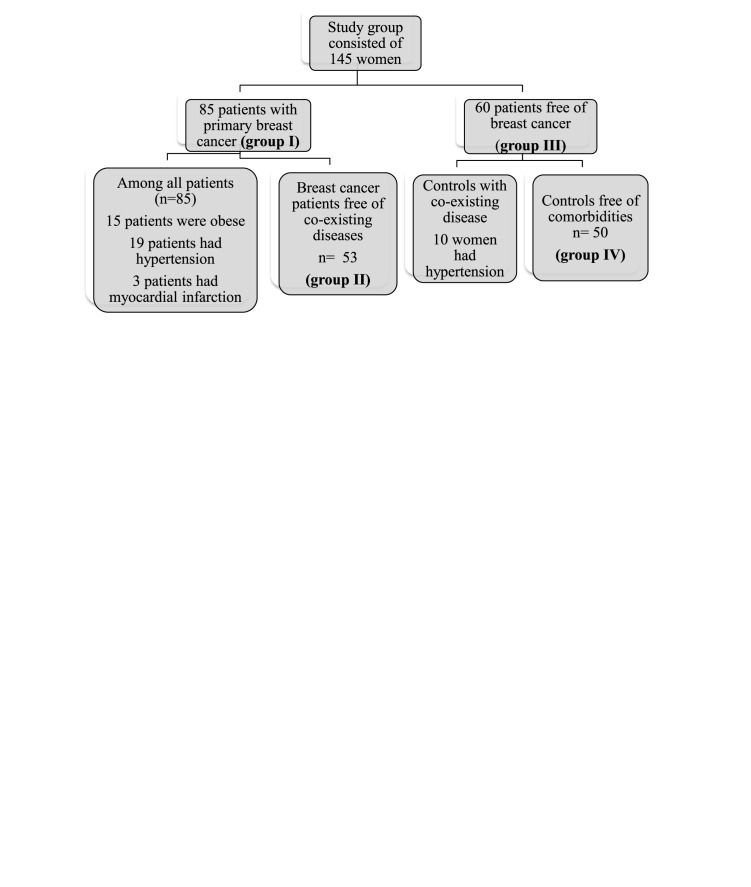
Patients’ characteristics with/without concomitant diseases

Between November 2015 and June 2017, 145 consecutive females were enrolled in the study. Eighty-five were patients with primary BrCa (M0), the median age of diagnosis was 55 years and within this group, 15 patients were obese, 19 patients had hypertension, and 3 patients had previous myocardial infarction event (group number I). After adjustment of coexisting diseases from the investigation group, we reached a homogeneous BrCa cluster (group number II). Histological grading and IMH ER/PR/HER2/Ki67 evaluation were measured in this cohort of patients using standard criteria and procedures. Fifty-eight were diagnosed with luminal-A type BrCa, 88% of patients had invasive breast ductal carcinoma. Population characteristics are summarized in Table 1. Another group consisting of 60 women free of BrCa were enrolled in the study as controls (group number III). Among those females, ten had hypertension, so after exclusion of hypertensive women, we created group number IV named: subjects free of BrCa and coexisting disease (Figure 1).

Significantly higher concentrations of TF and lower TFPI concentrations were noted in the study group as compared with controls, regardless of coexisting obesity and cardiovascular diseases (*P*<0.0001 respectively). Based on TF and TFPI concentrations, TF/TFPI concentration ratios were calculated and we observed a seven-fold higher procoagulant ratio in BrCa cases. However, in terms of TF and TFPI activity, only a significantly higher activity of TF and a lower activity of TFPI in the BrCa subjects free of comorbidities as compared with perfectly matched healthy post-menopausal women (*P*=0.0049, *P*=0.0379 respectively) were noted. Consequently, TF/TFPI activity ratio was higher, which may indicate a shift towards overactivation of coagulation cascade in those patients (*P*<0.0064) (Table 2).

**Table 2:** Plasma levels of TF activity and concentration, (TF) pathway inhibitor activity and concentration (TFPI), TF/TFPI activity and concentration ratios, t-PA, PAI-1 in patients with BrCa as well as in subjects free of BrCa depending on coexisting diseases

Coagulation assays (units)	BrCa patients	BrCa patients free of coexisting disease	Subjects free of BrCa	Subjects free of BrCa and coexisting diseases	*P*-value			
	Group I	Group II	Group III	Group IV	I vs II	I vs III	I vs IV	II vs IV
**TF activity** (pM)	14.01	16.29	13.10	12.69	*P*=0.2358	*P*=0.0938	*P*=0.0767	***P*=0.0049**
	11.17/25.56	12.31/29.18	10.36/18.59	10.36/18.45				
**TF concentration** (pg/ml)	535.40	535.40	116.65	116.65	*P*=0.9359	***P*<0.0001**	***P*<0.0001**	***P*<0.0001**
	400.86/710.40	417.04/670.18	90.90/183.44	90.90/167.61				
**TF/TFPI activity ratio**	10.79	15.42	10.78	10.20	*P*=0.2615	*P*=0.1741	*P*=0.1054	***P*=0.0064**
	7.48/25.81	9.15/28.93	7.19/14.91	6.86/14.45				
**TFPI activity** (U/ml)	1.32	1.23	1.30	1.57	*P*=0.5449	*P*=0.3201	*P*=0.1574	***P*=0.0379**
	1.10/1.50	1.02/1.40	0.90/1.78	0.90/1.81				
**TFPI concentration** (ng/ml)	44.58	43.80	77.18	75.53	*P*=0.6422	***P*<0.0001**	***P*<0.0001**	***P*<0.0001**
	37.12/60.32	35.94/59.88	67.85/84.18	67.85/84.18				
**TF/TFPI concentration ratio**	10.76	11.50	1.56	1.56	*P*=0.7881	***P***<**0.0001**	***P***<**0.0001**	***P*<0.0001**
	8.67/17.05	8.72/18.49	1.12/2.67	1.17/2.49				
**t-PA concentration**(ng/ml)	5.19	4.97	4.59	4.54	*P*=0.7345	*P*=0.3593	*P*=0.2859	*P*=0.5434
	3.94/6.70	3.89/6.39	3.12/6.42	2.99/6.29				
**PAI-1 concentration** (ng/ml)	37.38	38.54	35.32	35.14	*P*=0.7421	*P*=0.2565	*P*=0.1914	*P*=0.1604
	27.87/47.43	27.08/50.57	21.17/51.51	21.17/49.62				

Significant differences between groups are denoted by bold *P*-values.

Apart from comparative analysis of the study and control groups, we have made statistical calculations in the investigation group based on menopausal status. An essential growing tendency towards a higher concentration of TFPI (*P*=0.0853) and a significantly higher concentration of t-PA were noted in post-menopausal BrCa patients as compared with pre-menopausal cases (*P*=0.0092). This analysis demonstrates that menopausal status indeed influences haemostatic profile expressed by an increase in the concentration of TFPI and t-PA. This phenomenon is associated with possible fibrinolysis activation and lower risk of thrombotic event in post-menopausal cases (Table 3).

**Table 3:** Concentrations and activities of selected haemostatic parameters in BrCa patients depending on menopausal status

Coagulation assays (units)	Pre-menopausal BrCa patients	Post-menopausal BrCa patients	*P*-values
	*n*=27	*n*=58	
**TF activity** (pM)	15.20/11.62/25.06	12.96/10.73/28.26	0.6760
**TF concentration** (pg/ml)	539.93/400.86/626.30	542.92/401.72/727.53	0.7010
**TF/TFPI activity ratio**	12.59/7.76/25.41	10.72/7.30/25.97	0.5963
**TFPI activity** (U/ml)	1.34/0.94/1.51	1.31/1.13/1.52	0.6724
**TFPI concentration** (ng/ml)	40.68/30.24/59.60	46.92/39.80/60.34	0.0853
**TF/TFPI concentration ratio**	12.67/8.43/19.52	10.66/8.77/14.72	0.4226
**t-PA concentration** (ng/ml)	4.23/2.62/5.30	5.70/4.24/7.21	**0.0092**
**PAI-1 concentration** (ng/ml)	37.20/27.87/76.01	38.23/29.20/44.79	0.3215

Significant differences are denoted by bold *P*-values. The underlined *P*-value represents closeness to statistical signifcance.

Furthermore, we hypothesized that concentrations and activities of selected haemostatic parameters can vary according to clinicopathological determinants as well as cardiovascular diseases and obesity. We conducted two separate statistical analyses: the first was focused on all BrCa cases (*n*=85) wherein an interesting observation was made that the levels of haemostatic parameters are age-dependent. Initially, a significantly higher concentration of TFPI in BrCa patients over 55 years than in the younger patients (*P*=0.0029) was obtained. It is also worth emphasizing that a significantly higher procoagulant potential expressed by TF/TFPI concentration ratio was recorded in younger women with BrCa (*P*=0.0045), even though the concentration of TF did not differ significantly in those women. For the next analysis, we divided BrCa patients according to the localization of their tumour: left or right breast. A significantly higher concentration of PAI-1 was noted in patients with BrCa localised in the left breast (*P*=0.0291). Subsequently, we further divided the study group into two subgroups: the first group consisting of patients with invasive ductal carcinoma and the second group with invasive lobular carcinoma. We observed significantly lower activity of TFPI and significantly higher concentration of PAI-1 in the group of patients with invasive ductal carcinoma as compared with invasive lobular carcinoma (*P*=0.0248, *P*=0.0275 respectively). However, according to clinical classification, we divided the study group into pT1 and pT2 subgroups and noted a significantly higher concentration of t-PA in patients with pT2 BrCa in relation to pT1 cases (*P*=0.0325) (Table 4).

**Table 4:** Concentrations and activities of selected haemostatic parameters depending on clinicopathological determinants in all patients from the study group (*n*=85)

Coagulation assays	TF activity	TF concentration	TF/TFPI activity ratio	TFPI activity	TFPI concentration	TF/TFPI concentration ratio	t-PA concentration	PAI-1 concentration
	*P*-values	*P*-values	*P*-values	*P*-values	*P*-values	*P*-values	*P*-values	*P*-values
**Age** <**55 years**	14.99/11.62/25.06	571.26/472.58/747.90	11.02/8.28/26.25	1.34/1.06/1.0	40.68/31.96/49.64	14.17/8.89/20.92	4.61/3.92/6.06	38.52/27.73/57.86
**Age ≥55 years**	13.16/11.03/26.92	500.39/387.90/602.66	10.43/7.37/24.13	1.30/1.12/1.48	48.24/41.00/64.24	10.02/8.46/11.75	5.38/4.20/6.66	37.19/28.10/44.46
	*P*=0.7299	*P*=0.1320	*P*=0.7038	*P*=0.9800	***P*=0.0029**	***P*=0.0045**	*P*=0.3443	*P*=0.2377
**Localization of tumour: left breast**	14.69/10.41/30.18	564.06/420.25/702.60	9.73/7.12/30.49	1.37/1.10/1.58	46.68/38.54/62.34	10.62/8.34/17.78	5.41/4.23/6.78	43.24/33.83/53.79
**Localization of tumour: right breast**	13.88/11.86/25.19	491.16/392.64/654.26	11.02/8.77/22.43	1.20/1.10/1.46	43.00/34.34/55.00	10.77/9.36/16.11	5.11/3.44/6.25	35.61/24.18/42.91
	*P*=0.9147	*P*=0.5010	*P*=0.8424	*P*=0.2043	*P*=0.2098	*P*=0.6500	*P*=0.4354	***P*=0.0291**
**Ki67 ≤14 %**	13.88/11.03/25.56	520.76/387.68/726.12	11.02/7.49/26.25	1.28/0.98/1.48	44.80/38.00/60.16	10.37/8.77/15.07	5.10/3.45/7.12	37.19/27.30/44.61
**Ki67 ≥15%**	14.99/11.17/31.56	562.47/439.56/686.50	10.56/7.08/25.81	1.38/1.10/1.54	42.36/32.96/61.88	11.38/8.47/20.92	5.30/4.23/6.70	39.50/28.10/53.01
	*P*=0.9319	*P*=0.5235	*P*=0.5875	*P*=0.1438	*P*=0.6731	*P*=0.4333	*P*=0.5669	*P*=0.3103
**Clinical classification TNM - pT1 (a, b, c)**	15.09/11.59/25.56	530.35/400.86/718.70	11.40/8.12/29.81	1.29/0.97/1.58	44.74/36.28/61.64	10.97/8.47/17.58	4.59/3.44/6.25	38.65/28.10/54.56
**Clinical classification TNM - pT2**	13.04/9.58/25.26	540.65/396.79/652.06	10.17/7.22/20.38	1.33/1.20/1.45	44.32/38.96/57.96	10.67/9.11/14.82	5.94/4.69/7.25	36.85/26.14/43.25
	*P*=0.3463	*P*=0.8286	*P*=0.2534	*P*=0.6887	*P*=0.8753	*P*=0.9525	***P*=0.0325**	*P*=0.1499
**Histological type IDC**	14.59/10.82/25.38	540.65/390.27/682.33	11.40/7.62/27.12	1.31/1.08/1.48	44.36/37.38/60.26	10.68/8.72/15.97	5.19/3.97/6.68	38.54/30.29/48.86
**Histological type ILC**	12.31/11.65/13.32	539.93/444.32/728.94	8.73/7.37/10.26	1.56/1.28/1.60	44.48/35.60/53.92	11.62/9.77/20.18	5.26/4.14/7.16	28.10/22.66/35.12
	*P*=0.4610	*P*=0.5055	*P*=0.1606	***P*=0.0248**	*P*=0.7693	*P*=0.5521	*P*=0.7154	***P*=0.0275**
**Luminal-A type of BrCa, *n*=58**	13.79/10.82/25.19	530.85/400.90/698.45	10.79/7.39/24.77	1.27/1.10/1.49	46.42/36.70/59.88	10.76/8.47/16.44	4.82/3.65/6.50	36.36/27.58/45.23
**Other molecular types of BrCa, *n*=27**	19.62/11.86/38.98	563.96/439.56/718.70	16.21/8.28/30.99	1.40/1.22/1.54	41.00/34.36/61.64	11.97/8.89/19.52	5.71/4.34/6.70	40.44/36.46/49.60
	*P*=0.1264	*P*=0.6939	*P*=0.4524	*P*=0.1422	*P*=0.3904	*P*=0.4047	*P*=0.4998	*P*=0.1568

Data are expressed as median (Me) and the IQR (lower quartile (Q1, upper quartile (Q3)); TNM classification of malignant tumours. Abbreviations: IDC, invasive ductal carcinoma; ILC, invasive lobular carcinoma.

Significant differences are denoted by bold *P*-values.

The second analysis was made only in patients with BrCa without comorbidities (Table 5). In the homogeneous BrCa group, depending on clinicopathological parameters, new results were obtained; a significantly higher TFPI concentration in BrCa patients over 55 years than in younger patients was recorded (*P*=0.0160). At the next step, we split BrCa patients into two subgroups in view of their expression of Ki67. The first group consisted of women with expression of Ki67 antigen ≤14% and the second group with Ki67 expression ≥15%. A markedly higher concentration of t-PA was observed in patients with expression of Ki67 higher than 15% (*P*=0.0370). Additionally, a significantly higher TFPI concentration was observed in luminal-A type BrCa as compared with patients having other molecular types of BrCa, including luminal B HER positive or negative, triple-negative and HER+/non-luminal (*P*=0.0396). Ultimately, we observed similar differences in the BrCa group without obesity and cardiovascular diseases, just as in all BrCa patients, in terms of localization and according to clinical classification (*P*=0.0250; *P*=0.0375 respectively).

**Table 5:** Concentrations and activities of selected haemostatic parameters depending on clinicopathological parameters in BrCa patients free of coexisting diseases (*n*=53)

Coagulation assays	TF activity	TF concentration	TF/TFPI activity ratio	TFPI activity	TFPI concentration	TF/TFPI concentration ratio	t-PA concentration	PAI-1 concentration
	*P*=values	*P*=values	*P*=values	*P*=values	*P*=values	*P*=values	*P=*values	*P*=values
**Age <55 years**	14.99/11.59/24.17	564.16/383.00/649.85	11.02/8.28/25.41	1.28/0.94/1.42	39.00/31.92/46.44	14.02/8.43/20.36	4.55/3.92/5.98	37.20/26.42/56.19
**Age ≥55 years**	25.72/13.24/35.28	542.92/441.94/698.45	21.46/10.02/29.95	1.26/1.13/1.44	47.58/40.92/66.62	10.68/9.57/12.23	6.09/3.72/7.06	38.47/24.29/46.37
	*P=*0.0712	*P=*0.5757	*P=*0.2217	*P=*0.8640	***P=*0.0160**	*P=*0.2987	*P=*0.3204	*P=*0.6560
**Localization of tumour: left breast**	23.14/11.38/37.64	566.31/458.45/670.18	17.11/7.88/31.58	1.34/0.99/1.41	44.74/36.96/62.28	11.50/8.19/20.22	4.93/4.05/6.65	44.19/32.28/57.98
**Localization of tumour: right breast**	13.96/12.46/26.92	500.39/392.64/710.40	11.54/10.09/22.43	1.20/1.10/1.50	40.68/32.96/50.80	11.75/9.77/18.46	5.26/3.92/6.34	35.12/22.22/39.39
	*P=*0.5315	*P=*0.3006	*P=*0.5466	*P=*0.6008	*P=*0.4460	*P=*0.9004	*P=*0.8112	***P*=0.0250**
**Ki67 ≤14 %**	19.79/12.46/28.76	530.35/383.00/649.85	15.11/9.95/29.99	1.23/0.84/1.34	44.74/39.00/64.24	10.49/9.36/14.02	4.35/3.24/6.25	38.54/24.18/48.12
**Ki67 ≥15%**	18.21/11.59/31.56	563.96/444.32/686.50	16.21/8.65/25.81	1.36/1.14/1.42	40.12/31.96/59.60	12.67/8.65/21.60	5.87/4.42/6.86	36.53/26.42/53.01
	*P*=0.7329	*P*=0.4193	*P*=0.5618	*P*=0.2953	*P*=0.1138	*P*=0.0901	***P*=0.0370**	*P*=0.9909
**Clinical classification TNM - pT1 (a, b, c)**	21.16/12.31/29.59	547.45/402.49/653.85	16.54/8.73/29.99	1.28/0.84/1.42	43.84/36.28/64.24	11.07/8.65/17.05	4.44/3.24/6.34	41.15/26.42/57.65
**Clinical classification- TNM - pT2**	13.88/12.31/26.92	563.96/431.59/728.94	14.57/10.09/20.49	1.26/1.20/1.38	42.36/35.60/46.44	12.67/9.77/20.36	5.87/4.83/6.44	36.50/24.40/38.56
	*P*=0.5395	*P*=0.6253	*P*=0.6077	*P*=0.9601	*P*=0.4754	*P*=0.4015	***P*=0.0375**	p = 0.1723
**Histological type- IDC**	18.92/12.39/29.18	559.47/401.68/651.85	16.54/9.19/29.95	1.28/1.02/1.41	41.54/35.32/60.74	11.25/8.54/18.28	5.20/3.93/6.65	38.54/25.45/54.60
**Histological type- ILC**	12.82/11.80/26.92	613.22/520.76/728.94	10.17/8.73/18.68	1.30/1.20/1.60	44.32/35.60/44.80	15.04/11.38/20.18	5.05/4.14/6.16	30.24/24.18/35.12
	*P*=0.4518	*P*=0.2969	*P*=0.2969	*P*=0.3832	*P*=0.8241	*P*=0.3832	*P*=0.9864	p = 0.1660
**Luminal A type of BrCa (*n*=38)**	14.10/12.31/26.92	539.93/431.59/653.85	12.16/9.73/25.81	1.26/1.10/1.40	44.80/37.12/60.16	10.75/8.77/17.05	4.55/3.45/6.22	38.56/28.10/45.48
**Other molecular types of BrCa (*n*=15)**	23.14/14.18/39.16	547.41/439.56/718.70	18.80/8.98/30.99	1.38/1.14/1.42	38.36/32.96/43.44	13.71/8.89/20.92	5.79/4.42/6.86	38.35/27.73/53.01
	*P*=0.1636	*P*=0.9243	*P*=0.3926	*P*=0.3422	***P*=0.0396**	*P*=0.1424	*P*=0.3161	p = 0.6806

Data are expressed as median (Me) and the IQR (lower quartile (Q1, upper quartile (Q3)); TNM classification of malignant tumours. Abbreviations: IDC, invasive ductal carcinoma; ILC, invasive lobular carcinoma.

Significant differences are denoted by bold *P*-values.

In the next stage of statistical analysis, correlation coefficients in all BrCa patients were calculated. Positive correlations were reported between TFPI and age as well as between TFPI with BMI (*P*=0.0018; *P*=0.0271 respectively). Additionally, a positive association between t-PA and age was noted (*P*=0.0311), whereas a negative correlation between TF activity and age was observed (*P*=0.0464). A positive correlation was identified between TF and addressing nodes as well as between TFPI activity and HER2 (*P*=0.0321; *P*=0.0387 respectively). However, a positive correlation between TF activity and histological grading as well as a negative correlation between TFPI activity and histological grading according to the Elston**–**Ellis classification in all BrCa cases were obtained (*P*=0.0332; *P*=0.0404 respectively) (Table 6).

**Table 6:** Correlations of haemostatic parameters with selected antropoclinicopathological parameters in all patients with BrCa (*n*=85)

Coagulation assays	TF activity R/*P*-values	TF concentration R/*P*-values	TFPI activity R/*P*-values	TFPI concentration R/*P*-values	t-PA concentration R/*P*-values	PAI-1 concentrationR/*P*-values
**Age (years)**	–0.2206/0.0464	–0.1501/0.1783	0.0046/0.9673	**0.3392/0.0018**	**0.3162/0.0311**	–0.1816/0.1024
**BMI (kg/m^2^)**	–0.0867/0.4414	0.0345/0.7600	–0.0400/0.7232	**0.2457/0.0271**	0.0788/0.4846	0.0731/0.5164
**Parity**	–0.0415/0.7113	–0.0927/0.4076	–0.0319/0.7757	0.0561/0.6169	–0.0020/0.9860	–0.0160/0.8863
**Diameter of the tumour (cm)**	0.0707/0.5609	0.1172/0.3340	0.0420/0.7298	0.0245/0.8406	0.1158/0.3396	0.0422/0.7288
**Histological grading**	**0.2994/0.0332**	–0.0999/0.4037	**– 0.2715/0.0404**	–0.0387/0.7468	–0.0258/0.8299	0.1313/0.2717
**Addressing nodes**	–0.0506/0.6645	**0.2906/0.0321**	0.0111/0.9243	–0.1115/0.3376	0.0970/0.4047	–0.0762/0.5130
**Ki67 expression**	0.0742/0.5242	0.0344/0.7681	0.1044/0.3696	-0.0302/0.7959	0.0700/0.5481	0.1592/0.1695
**E-cadherin**	–0.0157/0.8927	0.0617/0.5965	0.0460/0.6933	0.0133/0.9092	–0.1718/0.1379	–0.1936/0.0939
**HER2**	–0.1499/0.1962	–0.1312/0.2584	**0.2991/0.0387**	0.0413/0.7232	–0.0106/0.9277	0.1455/0.2099
**PR**	0.0601/0.6087	–0.26810.0511	–0.0206/0.8610	–0.1296/0.2677	0.0150/0.8983	–0.0190/0.8717
**ER**	0.0316/0.7867	–0.21550.0605	–0.0733/0.5289	–0.1726/0.2677	0.0427/0.7142	–0.0065/0.9556

Abbreviation: Parity, number of full-term pregnancies.

Significant differences are denoted by bold *P*-values.

Finally, from all BrCa patients, we selected subjects free of coexisting diseases and by doing so, we reached a homogeneous BrCa group (*n*=53) (Table 7). In this group, we reported additional associations. Firstly, a positive correlation was noted between TFPI concentration and age as well as between PAI-1 and BMI (*P*=0.0238; *P*=0.0247 respectively). Of significant interest is the fact that we noted a positive correlation between TF activity and histological grading as well as a negative correlation between TFPI activity and histological grading according to the Elston–Ellis classification (*P*=0.0236; *P*=0.0081 respectively). A negative correlation between TF concentration and the diameter of the tumour was found in BrCa cases without comorbidities (*P*=0.0168).

**Table 7:** Correlations of haemostatic parameters with selected antropoclinicopathological parameters in patients with BrCa without coexisting diseases (*n*=53)

Coagulation assays	TF activity R/*P*-values	TF concentration R/*P*-values	TFPI activity R/*P*-values	TFPI concentration R/*P*-values	t-PA concentrationR/*P*-values	PAI-1 concentration R/*P*-values
**Age (years)**	0.0839/0.5883	0.0759/0.6245	–0.1190/0.4417	**0.3869/0.0238**	0.2280/0.1366	–0.1381/0.3713
**BMI (kg/m^2^)**	0.0562/0.7203	–0.0398/0.8000	–0.1284/0.4119	0.1344/0.3900	0.0597/0.7036	**0.3904/0.0247**
**Parity**	0.0090/0.9537	0.0428/0.7828	–0.0084/0.9567	0.2434/0.1114	0.0366/0.8134	–0.0339/0.8270
**Diameter of the tumour (cm)**	-0.0358/0.8312	**– 0.4196/0.0168**	0.0493/0.7690	–0.0928/0.5797	0.1038/0.5351	–0.0242/0.8853
**Histological grading**	** 0.3850/0.0236**	–0.1272/0.4403	**– 0.4596/0.0081**	–0.1669/0.3097	0.0597/0.7179	0.1109/0.5017
**Addressing nodes**	–0.1657/0.2942	0.2698/0.0840	–0.0021/0.9893	–0.2061/0.1904	0.1211/0.4449	–0.1678/0.2880
**Ki67 expression**	–0.0550/0.7294	–0.0491/0.7574	0.1395/0.3783	–0.2141/0.1733	0.1984/0.2078	0.1002/0.5279
**E-cadherin**	–0.0114/0.9427	0.2021/0.1993	0.1030/0.5163	–0.1335/0.3994	–0.0496/0.7552	–0.1792/0.2560
**HER2**	–0.0511/0.7480	0.0000/1.0000	0.1387/0.3811	0.0726/0.6477	0.0733/0.6447	0.1515/0.3383
**PR**	0.0264/0.8684	–0.2029/0.1974	–0.1819/0.2490	–0.1660/0.2933	0.0237/0.8815	–0.1133/0.4749
**ER**	–0.0364/0.8190	–0.2460/0.0569	–0.0971/0.5408	–0.2153/0.1708	0.0030/0.9848	–0.0576/0.7170

Abbreviation: Parity, number of full-term pregnancies.

Significant differences are denoted by bold *P*-values.

## Discussion

In BrCa patients, interactions between haemostatic parameters and cancer biology is, to an extent, accepted as an important controller of BrCa spread [14]. Overactivation of the coagulation system has been noted in women with BrCa and is associated with cancer-mediated coagulopathy and, simultaneously, a lower overall survival and poor outcome in those patients. TF and its endogenous inhibitor, TFPI are two major players in the coagulation process, acting in an antagonistic manner, as well as in tumour biology where the opposite effect was also discovered [6,7]. The exact mechanisms by which these processes take place are still unknown and should, therefore, be explored in further detailed experiments [7,15,16].

Our study provides evidence that BrCa patients, regardless of coexisting diseases, not only display higher levels of TF protein but also lower levels of (TF) pathway inhibitor, resulting in a shift of the TF/TFPI ratio towards excess TF relative to healthy post-menopausal cases. After removing patients with coexisting diseases from the study group, we reached a homogeneous group and in those subjects, higher activity of TF and lower activity of TFPI were observed, consequently TF/TFPI activity ratio was higher as compared with healthy controls. This may suggest an elevated risk of thrombotic diathesis in BrCa cases. Hernández et al. [17], studied the level of TF in patients with various types of cancer, including BrCa and also found higher TF levels in their study group as compared with controls. However, they did not observe any relationship between the concentration of the TF and the risk of thrombosis. On the other hand, they emphasized that the increase in TF concentration is associated with a worse prognosis in those patients [17]. flTF stimulates intracellular signalling by binding to protease-activated receptors 1 and 2 (PAR-1 and PAR-2) that promote identified tumour-associated reactions [6,10,16]. TF overexpression has been shown to correlate with tumour progression, poor prognosis and decreased overall survival. Hypercoagulability may influence on malignant transformation, tumour growth, tumour cell–host interactions, and metastatic potential by stimulation of new vessels formation from pre-existing ones [9,11,12,15]. On the other hand, a lower concentration of TFPI in BrCa patients may be due to the fact that TF inhibitor is consumed in response to the elevated TF concentration as a compensatory mechanism. Because pro-coagulant TF activity is eliminated by TFPI, it also affects the reduction in proliferative mechanism together with inhibition of angiogenesis and metastasis [11].

In our study, we analysed BrCa patients according to the molecular nature of cancer and observed only higher concentrations of TFPI in luminal-A type as compared with other molecular types of BrCa, however only in the study group without comorbidities this observation was noted. Tinholt et al. [7] reported that the plasma levels of total TFPI tended to be lower in larger tumours, triple-negative tumours and grade-3 tumours. These results indicate that TFPI is inversely related to the ability of invasion and metastasis of BrCa and may also confirm that luminal-A BrCa presents the possibility of a more favourable outcome for patients in view of the non-haemostatic; tumour-suppressive activities of TFPI and anti-angiogenic and antimigratory properties of TFPI.

Apart from the regular nature of cancer expressed by oversecretion of TF and suppression in realization of TFPI, our study revealed an interesting phenomenon in all patients from the study group; the levels of haemostatic parameters are aged- and menopausal-status dependent. It is worth emphasizing that more than half the patients (56%) were aged 55 or more and 68% of patients were post-menopausal. The review of applicable literature indicates that as age increases, the risk of thromboembolic events increases. This is related to an elevated plasma pro-coagulant environment and fibrinolytic depletion. However, in our study, a significantly higher concentration of TFPI in BrCa patients over 55 years than in younger patients was observed irrespective of coexisting diseases. Our results occur in opposition to Ali et al. [18] who claimed that oestrogens directly down-regulate TFPI at the mRNA level, a process mediated by ERα and the genomic pathway. Our study, on the other hand, revealed that cancer, by its nature, presents sovereign regulation. Additionally, it should be noted that we recorded a significantly higher pro-coagulant potential expressed by TF/TFPI concentration ratios in all younger patients with BrCa even though the concentration of TF did not differ significantly in those women. In addition, Kocatürk et al. [10] and Hernández et al. [17] found no association between TF concentration and age in BrCa patients. Based on our findings, we speculate that younger women might have a higher risk of paraneoplastic thrombosis and worse prognosis because TF expresses non-coagulant functions in cancer biology by promoting tumour proliferation, angiogenesis activity and metastasis.

Furthermore, to support our hypothesis we calculated correlation coefficients and we observed positive correlation between TFPI concentration and age, regardless of comorbidities. Additionally, a positive association between t-PA and age as well as a negative correlation between TF activity and age were noted. Additionally, we conducted further analysis and noted a substantial growing tendency towards higher concentrations of TFPI as well as significantly higher concentrations of t-PA in post-menopausal BrCa patients as compared with pre-menopausal cases. Our findings suggest an opposite dynamic of BrCa biology dependent on age and hormonal status. Haemostatic profile could, therefore, be a relevant indicator of the cancer nature. Indeed, younger women with BrCa are more predisposed to cancer-related thrombosis, whereas, older women present enhanced tendency to fibrinolysis activation and better controlling TF-dependent blood coagulation. But, on the other side, higher expression of t-PA may contribute to tumour growth via extracellular matrix degradation followed by initiation of cancer cell migration [19].

It is worth emphasizing that TFPI has the ability to inhibit vascular endothelial growth factor (VEGF), plasminogenesis and metalloprotease, all of which have a significant effect on tumour growth and metastasis. Perhaps a higher concentration of TFPI is a compensatory mechanism in response to higher t-PA proliferative potential in older BrCa patients. In addition, Xu et al. [20] found a correlation between the degree of TFPI expression and the time of disease-free survival (DFS). Patients with higher expression exhibited longer DFS as compared with patients with lower expression or lack of expression. The authors also noted that lower expression patients are more likely to be at risk of recurrence [20].

Moreover, a significantly higher concentration of t-PA in patients with expression of Ki67 higher than 15% in relation to patients with Ki67 antigen below 14%, notwithstanding this relationship was obtained only in the study group without comorbidities. Ki67 is a proliferative activity marker applied as a marker of tumour aggressiveness and is in use to predict prognosis. Ki67 expression above 15% indicates a high mitotic index, being the percentage of cells that are in the mitosis phase, which in BrCa patients is an unfavourable factor. The potential of Ki67 expression demonstrates the tumour proliferation rate and correlates with the initiation, progression, metastasis and prognosis of BrCa [21]. t-PA leads to the development of metastasis through proteolytic degradation of the extracellular matrix. It is known that t-PA induces proliferation in a wide variety of cell types [19], including BrCa cells. Furthermore, according to clinical classification of the tumour, a significantly higher concentration of t-PA in patients with pT2 BrCa in relation to pT1 cases was noted, which is in line with Baluka et al. [19]. They observed higher t-PA concentrations in tissue homogenates in pancreatic cancer depending on the T (TNM) stage [19]. Taken together, those observations can be negative indicators due to the fact that t-PA is involved not only in the dissolution of cross-linked plasma fibrin, but also in extravascular proteolysis and tissue remodelling resulting in a pro-angiogenic profile enabling tumour invasion, cancer cell migration and spreading. Additionally, t-PA may indicate on metastatic phenotype and increased proliferation of tumour cells. All of these alterations directly contribute to enhanced cancer growth and aggressiveness [19]. A higher level of t-PA is related to poor prognosis in patients with BrCa.

Besides age and menopausal status, we cannot disregard another anthropometric parameter—BMI. We have observed interesting BMI relationships, first of all, a positive correlation between TFPI concentration and BMI in all BrCa patients, but also a completely different association in the case of BrCa patients after excluding those patients with coexisting diseases; in this group, we reported a positive correlation between PAI-1 and BMI. These correlations indicate the opposite tendency depending on the amount of adipose tissue and cardiovascular diseases. The BrCa group with other diseases also present an association; along with an increase in BMI, there is an increase in TFPI, which suggests that enlargement of adipose tissue enhances releasing TFPI and better limiting of coagulation. Nevertheless, in the homogeneous BrCa group, correlation indicates suppression of fibrinolysis expressed by an increased PAI-1 with BMI.

Tumour size, lymph node positive status, histological tumour type and histological grade are the most relevant prognostic factors of the clinical course of BrCa. In our study, we observed a positive correlation between TF activity and histological grading as well as a negative correlation between TFPI activity and also histological grading according to the Elston–Ellis classification. Tumours with higher histological grade have a more invasive character. This invasive phenotype may lead to overstimulation of pro-coagulant TF, severe increases in hypercoagulability and a decline in survival rate of patients with a higher tumour grade. However, Molnar et al. [22] studied the activity of TF in the plasma of patients with different types of cancer and did not receive any significant difference in TF activity and clinical stage. According to the authors, TF activity is not a useful marker for differentiation of cancer stage [22], the author’s statement is the reverse of our findings. However, Tinholt et al. observed reduced expression of *TF* mRNA in patients with G3-stage BrCa compared with women with G1 and G2 stages [7]. The authors suggest that the role of TF is more significant in earlier stages of tumour transformation. Additionally, authors underlined the clinical relevance of TFPI in BrCa. They observed a decrease in plasma TFPI levels with tumour size and the grade of malignancy, which is in-line with our findings [7]. Tinholt et al. [8] suggest that lower TFPI expression is associated with worse prognosis as well as lower survival rate and recurrence risk as compared with patients with higher TFPI expression. This is also due to the suggested use of TFPI as a marker for BrCa prognosis. They also point to the potential effect of TFPI as a BrCa suppressant [8]. However, our findings are in opposition to Stavik et al. [23] who claim that overexpression of TFPI is associated with tumour grade. Due to the TFPI decreases, the levels of antiproliferative, antimigratory, and anti-angiogenic mechanisms are abolished, resulting in increased tumour growth, malignancy and metastatic mechanisms [11].

In BrCa patients with coexisting diseases, a positive correlation between TFPI activity and HER2 positive status was reported. Oncogenic activation of human epidermal growth factor receptor-2 (HER2) and epidermal growth factor receptor (EGFR) enhances TFPI expression in BrCa [8]. TFPI acts in an inverse manner to TF and in the malignancy process, inhibits the cancer development [6]. It is important to underline that our study revealed interesting observations that activity of TFPI is more clinically relevant than TFPI concentration. The potential of TFPI activity tells us more about the ability of TFPI to inhibit the catalytic activity of the TF/VIIa complex to activate factor X to factor Xa. We speculate that TFPI presents dualistic functions and from one side might be an indicator of tumour expansion and invasive behaviour as well as simultaneously presenting antiproliferative properties and being tumour suppressant.

A negative correlation between TF concentration and tumour size was noted in the current study but this correlation was observed only in BrCa cases without coexisting diseases. This suggests that higher concentration of TF is presented in patients with smaller tumours. But on the other hand, a positive correlation between TF concentration and positive nodal status was observed in BrCa subjects, however with coexisting diseases. These results indicate that age-related diseases such as hypertension or obesity may influence the general clinical course of BrCa patients. Patients with coexisting diseases showing elevation of TF may suggest that TF is involved in tumour cells proliferation, which may indicate a role for TF in lymph node metastases (lymphatic spread). Our findings give us perspective to apply TF as a clinical relevant future prognosis marker. It can be used in screening for differentiation of BrCa, as well as helping in assessing tumour stage based on tumour size and nodal status. To sum up, an elevated level of TF is connected with increased cell motility and resistance to apoptosis which are two major components in metastasis and tumour growth.

Subsequently, after extracting two subgroups from the main study group, the first group consisting of patients with invasive ductal carcinoma and the second group of cases having invasive lobular carcinoma, we observed significantly higher levels of PAI-1 and lower TFPI activity in patients with invasive ductal carcinoma as compared with invasive lobular cancer patients, which is in line with Lampelj et al. [24] study. Our results may suggest that the histological type of BrCa is an indicator of fluctuations in haemostatic profile. Taken together, our opinion is that it is a negative predictor for invasive ductal carcinoma patients because it predisposes to a pro-coagulant environment expressed by simultaneous activation of coagulation and fibrinolysis suppression.

It is of great interest in our study we observed that localization of the tumour has relevant value in connection with haemostatic parameters due to the association with a markedly higher concentration of PAI-1 in patients with BrCa localized in the left breast. It is well-established that women are more likely develop cancer in the left breast, however, the reasons for left-right asymmetry in cancer are unknown; some research suggests that the left breast is usually larger than the right one. In the current study, the number of patients in each group was comparable (left breast = 43 compared with right breast = 42). Chen et al. emphasize the existence of ‘fluctuating asymmetry’ [25]. This is associated with slight differences in breast density, as some women are less tolerant to hormonal fluctuations during each menstrual cycle. The authors point out that asymmetry between right and left breasts may be associated with greater predisposition to BrCa development [13,25]. PAI-1 is well-characterized as a pro-coagulant, pro-inflammatory and profibrotic protein. Increased PAI-1 levels indicate ineffective fibrinolysis, hence this leads to increased thrombus formation [26]. Increased PAI-1 concentration is associated with activating signalling pathways that modify the tumour microenvironment and prevent apoptosis and promotion of angiogenesis, which boost malignant growth. PAI-1 stimulates angiogenesis via directly inhibiting proteases [6,15]. Clinically, elevated levels of PAI-1 correlate with poor relapse-free survival and poor overall survival in patients with BrCa. High levels of inhibitor PAI-1 have been associated with shorter survival rates in patients with BrCa [6]. Ferroni et al. [27] claim that elevated PAI-1 levels might be a prognostic marker of BrCa, and such observation coincides with our study. Lampelj et al. [24] claim that higher values of PAI-1 are usually noted in larger tumours, higher malignancy grades and invasive ductal histological types of BrCa. Our research shows that widespread understanding of the role of homeostasis in BrCa progression is still missing. However, more studies are needed to establish the true clinical relevance of our observations.

## Conclusion

Our findings confirmed that patients with M0 BrCa present a higher risk of cancer-related thrombotic events. Based on comprehensive analysis of haemostatic profile depending on clinicopathological features we speculate that haemostatic parameters play crucial roles in invasion and metastases of malignant tumours, hence, haemostatic profile may be used as an additional predictive indicator. Possibly, patients with luminal-A type of BrCa without comorbidities present better future outcomes due to higher concentration of TFPI. Definitely, haemostatic profile is age, localization and hormonal status dependent in BrCa cases.

## Abbreviations

BrCa: breast cancer
BMI: body mass index
BP: blood pressure
DFS: disease-free survival
ER: oestrogen receptor
FISH: fluorescence *in situ* hybridization
flTF: full-length tissue factor
HER2: human epidermal growth factor receptor 2
IHC: immunohistochemical
IMH: immunohistochemistry
Ki67: proliferation marker
PAI-1: plasminogen activator inhibitor type 1
PR: progesterone receptor
TF: tissue factor
TFPI: TF pathway inhibitor
t-PA: tissue plasminogen activator
VTE: venous thromboembolism
